# The effectiveness of the combined PBL and Tencent Conference online teaching mode in the clinical internship teaching of cardiac surgery

**DOI:** 10.1371/journal.pone.0315455

**Published:** 2024-12-19

**Authors:** Xiuwen Chen, Yao Xiao, Yunhui You, Jianxi Zhu, Shiqing Liu, Huiqiong Chen, Hong Zhu

**Affiliations:** 1 Teaching and Research Section of Clinical Nursing, Xiangya Hospital, Central South University, Changsha, China; 2 Xiangya Nursing School, Central South University, Changsha, China; 3 Department of General Surgery, Xiangya Hospital, Central South University, Changsha, China; 4 National Clinical Research Center for Geriatric Disorders, Xiangya Hospital, Central South University, Changsha, China; 5 Department of Rheumatology and Immunology, Xiangya Hospital, Central South University, Changsha, China; 6 Department of Respiratory Medicine, Xiangya Hospital, Central South University, Changsha, China; 7 Department of Cardiovascular Surgery, Xiangya Hospital, Central South University, Changsha, China; Touro University, UNITED STATES OF AMERICA

## Abstract

**Background:**

Educators increasingly emphasise the importance of clinical medical education reform, particularly the innovation of teaching models. Clinical internships in cardiac surgery are an essential stage in the development of medical students. Currently, it is still dominated by the traditional lecture mode. Therefore, exploring a new teaching model is a critical way to improve the quality of clinical internship teaching.

**Objectives:**

To investigate the effectiveness of the combined PBL and Tencent Conference online teaching mode in the clinical internship teaching of cardiac surgery.

**Methods:**

This historical controlled trial was conducted from September 2022 to January 2023. A total of 34 participants from the graduating class of 2020 took the combined PBL and Tencent Conference online teaching mode. 42 participants from the graduating class of 2021 adopted the traditional teaching method and were enrolled as a control group. All participants completed a questionnaire designed by the researcher to assess teaching quality. The questionnaire was filled out sequentially according to the chronological order of pre-internship, during the internship, and post-internship.

**Results:**

There was a statistically significant difference between the group of Tencent Conference online teaching based on the PBL mode and the group of traditional teaching mode in terms of the degree of participation (experience, hands-on opportunities, etc.), the degree of teacher-student interaction (questions, communication, etc.), and the degree of acceptance and understanding (key points, difficulties, and other learning concerns) in the class learning process during the internship (*p* < 0.05). The results of the survey also showed that there was a significant difference between the two groups in the scores of the degree of mastery and use of history taking in cardiac surgery and the degree of mastery and use of ancillary tests (application and interpretation of each test) after the internship (*p* < 0.05).

**Conclusions:**

The traditional approach currently used by most educators does not enable cardiac surgery students to transform theory fully. The PBL teaching method can stimulate students’ interest in learning and cultivate their comprehensive ability to a certain extent. Future educators should flexibly adapt the teaching method to the learning situation, develop a reasonable teaching mode of clinical internship, and ensure quality.

## Introduction

A clinical internship is an essential transition period for clinical students to combine theory with practical skills. It is also necessary to consolidate and improve basic theoretical knowledge as a preliminary basis for clinical diagnosis and treatment thinking. It plays a vital role in cultivating medical students’ literacy and ability. Cardiac surgery is a branch of significant surgery mainly focusing on cardiac macrovascular trauma, pericardial disease, heart disease, valve replacement, cardiac tumours, interventional treatment techniques, cardiac pacing and implantable defibrillation resuscitators, heart and lung transplantation, etc. The theoretical part of cardiac surgery for cardiac surgeons is abstract and difficult to understand because of the frequent morbidity and the extremely rapid changes in the conditions of patients. It is worthwhile for educators to explore how to transform obscure knowledge into a more intuitive understanding through clinical internships and improve the quality of teaching so that students’ experience is not superficial.

In recent years, recognising a fundamental need in contemporary education, integrating Information and Communication Technology (ICT) into teaching practice has become an indispensable part of higher education [1]. Against this backdrop, foreign countries have implemented blended learning (B-Learning) to adapt to the new situation. The B-Learning teaching mode combines information digital teaching and traditional teaching methods to achieve cross-regional joint teaching [2]. Most teachers in China still adopt the traditional teaching mode, i.e., lecture-based, which can impart a large amount of knowledge in a limited time. Still, students have limited comprehension and a lack of subjective initiative due to passive acceptance of knowledge, so their practical and innovative abilities are poor [3]. PBL (Problem-based learning) has been a new teaching model in China in recent years. It was created and proposed by Barrows at McMaster University in Canada. It refers explicitly to placing learning in complex and meaningful problem situations and allowing students to work in groups to solve complex and practical problems based on constructing and solving complex problems [4]. It is a student-centred teaching method guided by constructivist theory and is a form of student-centred education based on real-world problems [5].

On the one hand, the PBL mode stimulates student’s enthusiasm to acquire knowledge and helps consolidate knowledge [6]. On the other hand, it also develops students’ problem-solving abilities and helps medical students gradually transition to clinical practice [7]. Medical education increasingly emphasises student-centeredness and has begun exploring new teaching models to improve teaching quality. For example, the practical application of PBL is being promoted in the class to integrate theory into problem analysis while focusing on developing clinical thinking and practical skills to fully stimulate students’ spirit of innovation and exploration and improve teamwork skills. Therefore, the PBL teaching mode is used throughout the cardiac surgery curriculum to systematically strengthen students’ clinical thinking and practical skills to meet the need for cardiac surgery teaching reform [8].

Meanwhile, during the implementation of the PBL teaching mode, the design of problems and cases poses a more demanding challenge to teachers, who should have solid professional knowledge and be able to foresee various issues and situations arising in the teaching process. Ensure the smooth running of the whole teaching process and achieve the expected teaching effect [9]. Additionally, teachers need to cultivate positive teacher-student relationships and integrate motivational factors into the design of their online courses. By understanding and addressing the various incentives that drive student learning, educators can tailor their online courses to suit the needs and interests of each learner, ultimately enhancing the overall educational experience [10]. This study investigates the effectiveness of the combined PBL and Tencent Conference online teaching mode in the clinical internship teaching of cardiac surgery.

## Methods

### Study population

**Fig 1:**
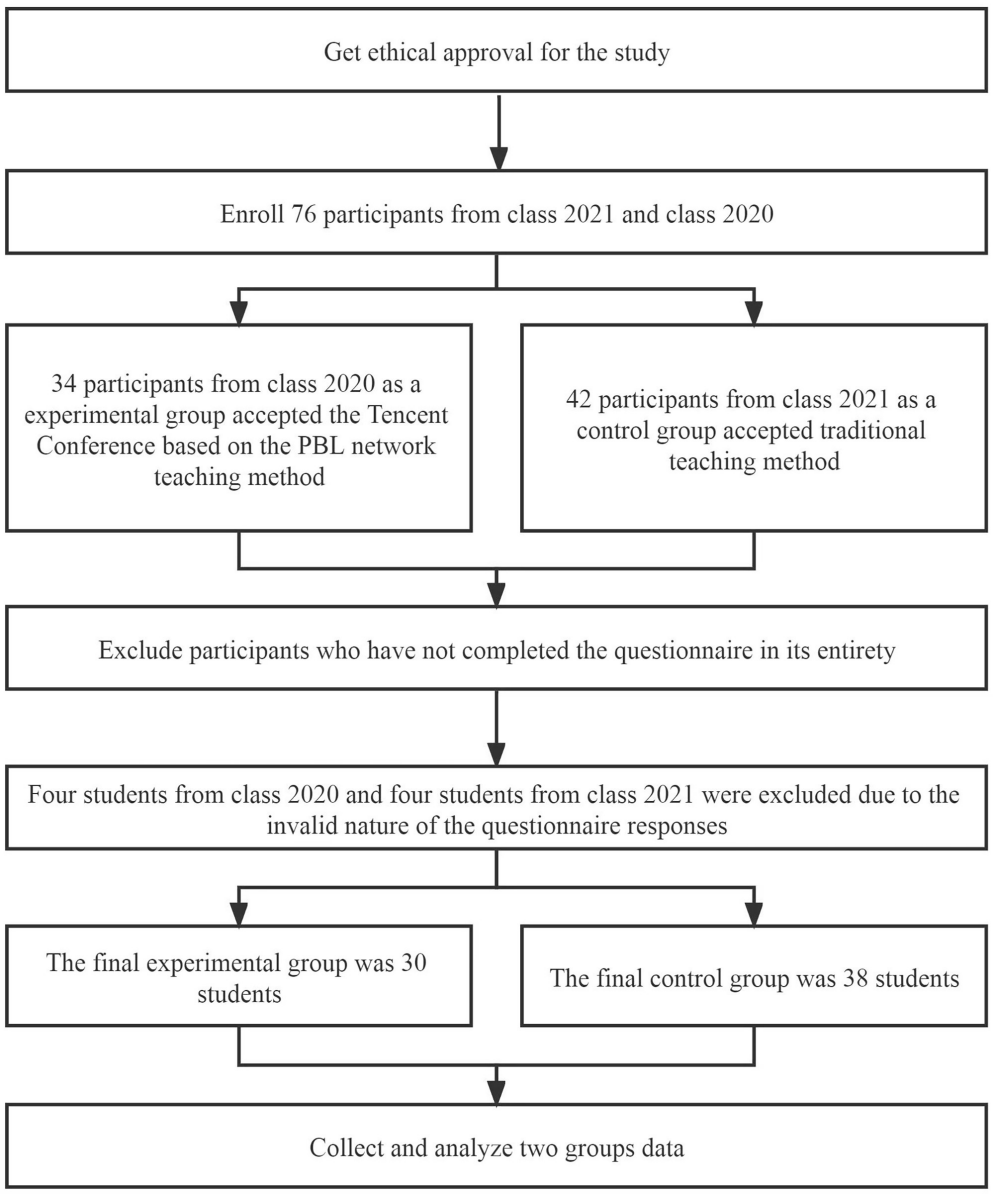
Grouping flowchart.

A convenience sample of clinical students from the graduating class of 2020 and the graduating class of 2021 was selected for this study. Inclusion criteria were: (a) undergraduate clinical students and (b) students with full-time undergraduate enrolment. Exclusion criteria were (a) dropouts or those with academic disabilities and (b) those who did not participate in theoretical or trial teaching. Clinical undergraduate students from the class of 2021 participated in the Tencent Conference web-based teaching course based on the PBL teaching method. For the control group, we had students from the graduating class of 2020 participate in courses with traditional teaching methods (i.e., students and teachers gathered in classrooms for a lecture-based and face-to-face conventional form of teaching) (Fig 1). The differing enrollment numbers in the two classes resulted in the participants of the two groups not being the same.

### Study design

This study has been reviewed and approved by the Clinical Medical Ethics Committee of Xiangya Hospital Central South University (reference:202204051) and was conducted following the Declaration of Helsinki. Consent indicates that if they engage and answer the survey form, their participation is willing and voluntary, often referred to as implicit consent. Besides, Participants’ anonymity will always be ensured, and the data collected will only be encoded into numbers.

This study was designed as a historical controlled experiment. Members of the graduating class of 2021 experienced 16 hours of traditional clinical cardiac surgery internship teaching methods, while the graduating class of 2020 took the same course information by adopting the Tencent Conference online teaching based on the PBL teaching mode. Both groups have the same number of class hours.

The curriculum of the PBL group was arranged as follows. Before the internship, the instructor asked students to organise themselves into groups; each group should have no more than 10 members. Each group chose a leader who would assign the group members to complete the PBL learning content. At the same time, the teacher prepared materials related to the course topic. The content includes a systematic review of relevant knowledge points (video), learning materials related to cases (documents), cases for discussion (3 cases), cases for simulated inquiry (1–2 cases), and extended cases (1–2 cases). Next, a group discussion is held without the teacher’s involvement before the internship. Each group would discuss one case, and each student would explain and describe the case (in the form of an oral presentation, PPT, or video). Then, the instructor organised the students to participate in an online discussion at the Tencent Conference. The analytical reflection and discussion of the case results were done in groups. Both students and the instructor can ask questions or make additions. The instructor provided a summary of the debate. Finally, the instructor made summative comments, offered targeted feedback, and assigned relevant assignments at the end of the class.

The traditional lecture group plan was as follows. Students were asked to preview the course before the apprenticeship briefly. Then, they had face-to-face learning and interaction through the conventional lecture-based classroom model, meaning that the instructor explained cardiac surgery knowledge in detail within a formal framework without dividing the class into small groups for discussion.

All participants completed the questionnaire. The whole questionnaire was carried out sequentially according to the student’s internship order. All students were given consent forms and were informed that their participation in the quizzes and surveys was voluntary. Also, the results of the quizzes and surveys had no (positive or negative) impact on the student’s course grades or performance. Participants’ private information is also kept strictly confidential.

### Hypotheses

The primary hypothesis was that there was a significant difference in learning effectiveness between clinical students in the PBL online teaching group and the traditional teaching group in the cardiac surgery internship. The secondary hypothesis was that there were no significant differences in course evaluations and in shaping the physician’s sense of professionalism between the two groups.

### Instruments

#### Demographic information questionnaire

According to the purpose of the research, the general information questionnaire was made, and the content included age, gender, grade point average (for the semester in which the internship took place), and the teaching method of the cardiac surgery clinical probation course.

#### Cardiac surgery teaching questionnaire

The research team prepared the questionnaire. After the research objectives are clearly defined and the data analysis plan is formulated, the design is focused on specific problems such as pre-teaching preparation, teaching practice, post-teaching feedback and performance tests. The questionnaire was adjusted and refined through brainstorming sessions with team members to ensure accuracy before the formal investigation. There are 24 items, each on a 10-point scale from 1 (low) to 10 (high). The total score includes four dimensions: self-learning ability before internship(3 items), learning conditions during the internship(4 items), satisfaction with the learning effects after internship(13 items), and shaping of physicians’ sense of professionalism after internship(4 items), with a total score of 240. The Cronbach’s alpha calculated from the collected data was 0.978, indicating the scale’s high reliability.

### Data evaluation and statistical analysis

Both groups of participants were asked to complete the pre-apprenticeship portion of the scale before starting the cardiac surgery internship course to assess the degree of independent learning and preceptorship. During the internship, students were asked to complete questions on class learning effectiveness and student-faculty interaction as essential indicators of academic performance in the class. After the internship, they were required to complete both the classroom satisfaction assessment and the sense of professionalism sections of the scale. To track each student’s learning path and effectiveness, the entire questionnaire was conducted item by item in chronological order.

The data were analysed using SPSS Statistics 26.0 (IBM Corp., Armonk, NY, USA). The normal distribution of the study variables was confirmed using the Shapiro-Wilk test *(p* > 0.05) and Kolmogorov-Smirnov test (*p* > 0.05). Frequency and frequencies with percentages were computed to describe the enumeration data, such as gender. Average standard deviations (S.D.) were used to describe the measurement data, such as the examination results for the semester in which the student was apprenticed. Independent samples t-test was used to compare the differences in student scale scores across teaching modes.

## Results

### Respondents characteristics

Seventy-six questionnaires were sent out, and 68 valid questionnaires were returned, with a response rate of 89.47%. 55.88% (n = 38) of the 68 respondents were male and 44.12% (n = 30) were female. The ages of the participants ranged from 21 to 25 years, with a median of 23 years and a mean of 23.16 ± 0.80 years. 44.12% (n = 30) of students took online and 55.88% (n = 38) of students took offline. The mean exam score in the semester where the online teaching internship was held was 80.37±6.21 (n = 30), and the mean score of the exam in the semester where the offline teaching internship was held was 77.05 ± 5.88 (n = 38). It can be concluded that this teaching method is flexible and attractive and focuses on the cultivation of student’s abilities and qualities, and the difference between the two groups is statistically significant (*p* < 0.05) (Table 1).

**Table 1:** Participants’ demographic characteristics.

Characteristics	PBL group	Traditional group	*p*
Group size	30	38	
Age (Years)	23.47±0.86	22.92±0.67	0.005
Gender (%)			0.112
Male	20(66.67)	18(47.37)	
Female	10(33.33)	20(52.63)	
Grade point average (for the semester in which the internship took place)	80.37±6.21	77.05±5.88	0.028

### Analysis of Internship of clinical cardiac surgery student’s with different teaching modes

Although there was no statistically significant difference between the two groups in the total score of cardiac surgery teaching (*p* = 0.065), the total score in the PBL group was 191.17±37.61, significantly higher than in the traditional group (175.11±33.00).

#### Comparison of two groups of self-learning ability assessment scales scores before internship

There was no significant difference in the student’s interest in learning cardiac surgery, the degree of understanding, or the degree of preparation for the knowledge and skills of related diseases before the internship. In addition, students scored significantly higher on their interest in learning than on comprehension and readiness (shown in Table 2).

**Table 2:** Comparison of two groups of self-learning ability assessment scales scores before internship (*N* = 68).

Variable	PBL group (N = 30)	Traditional group (N = 38)	*t*	*p*
Q1: Interest in Learning	7.97±1.47	7.21±1.81	1.848	0.069
Q2: Degree of Understanding	6.80±2.44	6.08±2.10	1.309	0.195
Q3: Degree of Preparation	6.87±2.45	6.50±2.04	0.675	0.502
Q1-Q3 Total score	21.63±5.88	19.79±5.30	1.358	0.179

#### Comparison of two groups of learning conditions during the internship

As can be seen from Table 3, there was a significant difference between the scores of the two groups in terms of the degree of participation (experience, hands-on opportunities, etc.), the degree of teacher-student interaction (questions, communication, etc.), and the degree of acceptance of understanding (key points, difficulties, and other learning concerns) of classroom learning during the internship (*p* < 0.05). In other words, the students who adopted PBL combined with the Tencent Conference online teaching mode had significantly higher classroom learning engagement scores, teacher-student interaction scores, and receptive understanding scores than those who adopted the traditional teaching mode. And there was no significant difference in student’s class concentration levels regardless of which teaching mode was adopted. Therefore, compared with the conventional teaching method, the PBL method can improve students’ learning initiative, knowledge mastery and analytical ability, and communication and teamwork ability.

**Table 3:** Comparison of two groups of the learning conditions during the internship (*N* = 68).

Variable	PBL group (N = 30)	Traditional group (N = 38)	*t*	*p*
Q4: Degree of Concentration	7.93±1.66	7.32±1.85	1.431	0.157
Q5: Degree of Participation	7.80±2.09	6.82±1.90	2.028	0.047** (p<0.05)
Q6: Degree of teacher-student interaction	8.07±1.95	7.11±1.84	2.084	0.041** (p<0.05)
Q7: Degree of Acceptance and Understanding	8.03±1.67	7.16±1.87	2.009	0.049** (p<0.05)
Q4-Q7 Total score	31.83±6.76	28.39±6.86	2.067	0.043** (p<0.05)

#### Comparison of two groups of satisfaction for lesson effectiveness and shaping a sense of professionalism after internship

The results of the mastery and utilisation degree scores of the two groups for cardiac surgery history taking and the mastery and utilisation degree scores of the ancillary tests (application and interpretation of each test) after the internship were significantly different (*p* < 0.05) (Table 4). The experimental group scored significantly higher than the control group. In contrast, there were no significant differences in the scores for the entries of satisfaction with overall teaching and impressions of surgical learning. In addition, there were no significant differences in the scores comparing the two groups on the physicians’ sense of professional shaping, as detailed in Table 5.

**Table 4:** Comparison of two groups of satisfaction with the learning effects after internship (*N* = 68).

Variable	PBL group (*N* = 30)	Traditional group (*N* = 38)	*t*	*p*
Q8: Evaluation of overall teaching satisfaction	8.43±1.48	8.05±1.69	0.973	0.334
Q9: Evaluation of overall cardiac surgery study impressions	8.23±1.61	7.71±1.64	1.314	0.193
Q10: Degree of mastery of clinical thinking in cardiac surgery	8.07±1.91	7.18±1.77	1.972	0.053
Q11: Degree of mastery and consolidation of the theoretical knowledge system of cardiac surgery	8.00±1.78	7.24±1.57	1.877	0.065
Q12: Degree of mastery and consolidation of specialised cardiac surgery practice	7.90±1.92	7.08±1.73	1.852	0.068
Q13: Degree of mastery and use of cardiac surgical history taking	8.47±1.59	7.37±1.73	2.691	0.009 (p<0.05)
Q14: Degree of mastery of the use of physical examination in cardiac surgery (in particular, the cardiac specialist physical examination: visual, tactile, percussive and auditory)	8.17±1.86	7.71±1.52	1.113	0.27
Q15: Degree of mastery and application of cardiac surgery auxiliary examinations (application and interpretation of each examination item)	7.87±2.08	6.95±1.61	2.055	0.044 (p<0.05)
Q16: Degree of mastery of diagnostic and differential diagnosis in cardiac surgery	7.80±1.77	7.03±1.70	1.830	0.072
Q17: Degree of mastery of the principles of cardiac surgical treatment protocols	7.83±1.88	6.97±1.87	1.881	0.064
Q18: Degree of mastery of common diseases and surgical procedures in cardiac surgery	7.40±2.25	6.53±2.06	1.665	0.101
Q19: Degree of awareness and attention to clinical, humanistic care (communication skills, etc.) in cardiac surgery	8.27±1.80	7.82±1.41	1.159	0.251
Q20: Degree of mastery of communication skills with cardiac surgery patients	8.07±1.95	7.63±1.51	1.037	0.303
Q8-Q20 Total score	104.50±21.83	95.26±18.61	1.882	0.064

**Table 5:** Comparison of two groups of scores on the shaping of physician’s sense of professionalism after internship (*N* = 68).

Variable	PBL group (N = 30)	Traditional group (N = 38)	*t*	*p*
Q21: Professional Honor & Responsibility	8.47±1.66	8.32±1.42	0.405	0.687
Q22: Sense of conviction and firmness of career	8.33±1.75	7.95±1.74	0.907	0.368
Q23: Interest in surgery	8.40±1.79	7.82±1.67	1.385	0.171
Q24: Interest in cardiac surgery	8.00±2.05	7.58±1.50	0.978	0.332
Q1-Q24 Total score	33.20±6.33	31.66±5.63	1.061	0.293

## Discussion

### Current status of domestic and international teaching models

This study shows that the average grades of students who adopted the PBL combined with the Tencent Conference teaching model in the semester they were interned were significantly higher than those of the traditional teaching model group (*p* < 0.05). It shows that implementing the PBL teaching mode can effectively improve the effect of internship and independent learning ability to a certain extent. At the same time, it also references the reform of clinical internship teaching.

The undergraduate level should enable students to acquire solid professional knowledge and clinical skills and improve their core competencies to adapt to workplace requirements as soon as possible. As one of the three main aspects of clinical undergraduate education, a clinical internship is a meaningful way to cultivate student’s core qualities and improve their overall quality. The internship model has perfected the practical teaching system of medical students’ early exposure to the clinic, zero connection between theory and practice, and a constant line between classroom and hospital [11]. The PBL method of medical education was introduced in the 1960s by Professor Barrow, a pioneer of medical education reform in the United States. It is a problem-based approach that allows students to master the scientific knowledge implicit behind the problem through learning around the issue, improving their ability to solve problems on their own and learn on their own, with the ultimate goal of developing lifelong learning habits. A core principle of PBL in undergraduate medical education is that basic science concepts are better understood, remembered, and applied when learned in a clinically relevant format. There is considerable variation in the implementation of PBL across medical schools. Still, at least 109 U.S. medical schools use this approach to some degree, and 37 schools use PBL as their primary teaching method [12].

The PBL teaching method has been widely used in Western medical teaching, but medical education reform is progressing in China. The traditional teaching model mainly adopted in China is teacher-centred and lecture-based, with theoretical knowledge imparted for the pathogenesis, clinical manifestations, diagnosis, treatment and prognosis of diseases, while medical students need to transition from theoretical understanding to clinical. The traditional teaching mode gradually shows inadequacy in transforming medical students into doctors. Students are too passive in teaching and lack the initiative, innovation, and enthusiasm to think about problems. However, in recent years, China’s cardiac surgery department has skillfully combined ultrasound technology with PBL mode so that students can grasp the essence of cardiac ultrasound technology more effectively; instead of simply listening to the teacher’s lecture, they can master this technology exactly and can be integrated into clinical practice, so that students can learn ultrasound examination skills and cultivate ultrasound diagnostic thinking by searching for information and analysing cases, and also review The student’s learn ultrasound skills and develop ultrasound diagnostic thinking by searching for information and analysing cases, and also review and consolidate basic knowledge of anatomy, pathology and pathophysiology [13]. It allows students to exercise their analytical skills and review the literature in proposing and solving problems, thus expanding their knowledge, broadening their horizons, and increasing their participation, making learning and internship more flexible and active [14, 15].

### Effect of different teaching modes on the effectiveness of internship

#### Pre-internship

The results showed no significant difference in the independent learning ability scores between the two groups before the internship. The current theoretical knowledge of cardiac surgery only through lectures makes the teachers not pay enough attention to teaching and providing feedback to students after class, so students can easily forget the knowledge points of instruction and cannot further understand and apply them. In addition, the clinical internship phase of surgery is also primarily a repetitive lecture of classroom theory, which is still teacher-driven, and students’ willingness to review in advance and their motivation to learn is not high.

With the continuous changes in medical education, the traditional education concept has been gradually replaced by the new education concept of "student-centred and teacher-led" so that the key to the change of modern teaching concept is to focus on cultivating student’s independent learning ability [16]. The cultivation of independent learning ability is the core element that promotes the overall development of medical undergraduates. Thus, educators should change the traditional teaching methods, give full expression to the student’s leading position in the classroom, and let them become the classroom master [17]. In this way, students can fully mobilise their learning enthusiasm and prepare for class, improve their independent and logical thinking ability, develop good learning habits and improve learning efficiency.

#### During the internship

The study showed that the group that adopted the PBL combined with Tencent Conference online teaching mode during the internship had significantly higher scores for the degree of participation in cardiac surgery learning, the degree of teacher-student interaction (questions, communication, etc.), and the degree of acceptance of understanding (key points, difficulties, and other learning concerns) than the group that adopted the traditional mode of teaching (*p* < 0.05). This is because the conventional internship teaching model played the leading role of teachers but mainly imparted knowledge and neglected the development of student’s abilities. Edgar’s “learning pyramid” theory suggests that discussion, practice, and demonstration retain 50%, 75%, and 90% of the content, respectively, while listening and reading retain only 20% and 10%, respectively [18]. Moreover, with the increase in medical students’ grade levels, the accumulation of knowledge and the development of medical technology, the limitations of the traditional teaching model for medical students’ practical operation, case analysis and doctor-patient communication are becoming more and more prominent. The continuous exploration and practice of new teaching models are more urgent [19]. In addition, the cardiac surgery didactic curriculum has more circular content. Therefore, educators should highlight the key points and grasp the principles of conciseness, simplicity, and systematicity in the course of the short internship time [20]. They can select the teaching contents and implement the principle of less but more precise teaching according to the syllabus requirements. In the teaching process, the focus is on standard and multiple diseases.

In contrast, compared with the traditional teaching method, the PBL model enables medical students to learn clinical reasoning thinking, improve innovation, reflect teamwork, strengthen expression skills, and acquire communication and interpersonal skills [21]. It is also characterised by the ability to exercise students’ learning and thinking to find solutions to problems, develop and exercise their diagnostic imaging thinking, and develop collaboration and resilience. Second, due to the COVID-19 epidemic, traditional education has swiftly transitioned to online education, posing an unmistakable emergency for teachers and students. This sudden shift has markedly increased the importance of technological integration in education, compelling teachers to update and enhance their teaching capabilities to adapt to the new environment [22]. In this context, the Tencent conference was used as a carrier combined with the PBL method to adopt an online teaching model in this study [23]. This provides a broad research perspective for numerous educators and opens up a new platform for classroom and practical teaching [24].

*Post-internship*. The current study showed that the experimental group’s scores for the mastery and use of cardiac surgical history taking and ancillary examinations after the internship were significantly higher than those of the control group (*p* < 0.05). This indicates that the PBL teaching method can stimulate interest in learning and substantially improve students’ ability to analyse and solve problems. It is conducive to developing medical students’ clinical questioning and body-checking skills. However, the inability to contact patients in wards and observe clinical operations on-site deprives students of valuable opportunities for clinical consultation, body examination and hands-on practice, which may negatively impact their future clinical practice.

On the other hand, although there was no significant difference in the scores on shaping physicians’ sense of professionalism, this may be due to the insufficient length of the experiment or the small sample size. In any case, the internship should focus on developing students’ communication skills, ability to write medical records, and learning medical and legal provisions to build a sense of professionalism and ethics as a physician. Physician professionalism is not innate but is determined by factors such as life environment, education level, and engagement in practice activities. Therefore, nurturing medical students through scientific and rational ways and means will contribute to the construction of physician professionalism [25].

### Education reflection and prospect

Implementing online teaching for surgical internships is both a challenge and an opportunity for educators. The online teaching model is an inevitable trend in the development of education informatisation and modernisation, which helps to integrate high-quality educational resources and improve the quality of education. In the present day of information technology development, the rapid growth of networks gradually emerged diversified teaching modes; the analysis, combination and active practice of these teaching modes for the teaching reform of cardiac surgery internship has essential significance. According to the different teaching contents, combined with the analysis of the learning situation, teachers can flexibly and organically use these methods individually or in combination, gradually forming characteristic teaching methods that make trainees’ interest high and learning effect suitable. Through diversified teaching modes, we promote the collision and integration of trainees’ clinical thinking and modern theories, help them establish a scientific and innovative spirit, and cultivate creative and application-oriented talents. Mike Leland, a psychologist at Harvard University, first introduced the term "competency" in 1973, emphasising that "competency" is a personal characteristic that can distinguish the level of performance in a specific job and organisational environment [26]—using diversified teaching models, such as combining PBL with online teaching, to enhance job competency while developing student’s creative thinking, so that they can better adapt to the professional environment.

Cardiac surgery knowledge points are obscure and not easy to understand, and the online teaching model of PBL combined with Tencent meetings may not fully exploit its potential despite its convenience because the student needs more hands-on practice and on-site observation in a clinically oriented self-directed learning environment. Furthermore, in PBL-intensive programs, medical students may not learn all the information that primary science teachers consider most important, or they may misunderstand medical diagnosis or treatment because of the limited feedback they receive from their teachers. Similarly, Yeo S et al. showed that in the teaching model of PBL, insufficient time for student self-learning, low motivation, and insufficient typical cases all affect the successful implementation of PBL [27]. It lacks a holistic character, focusing on the patient’s problems without a holistic analysis of the patient, which can easily lead to a one-sided approach to problem identification. Interactive teaching is flexible and can be used to improve the effectiveness and quality of teaching in cardiac surgery clinical teaching [28]. For example, through the theme exploration type of interaction, the theme is used as a port, and the interactive teaching is carried out from the selected theme [27]. In addition, the PBL teaching mode also requires teachers to prepare well before the class, read a lot of relevant knowledge, and organise a logical sequence of questions, outlines, etc., to train the teachers and improve the overall quality of students at the same time.

Although the online teaching model of Tencent Conference based on the PBL teaching method used in this study has certain limitations, it is worth further exploration and practice as a promising teaching model with particular advantages in enhancing students’ learning ability, teamwork ability, and clinical practice.

### Limitations

The present study still suffers from some unavoidable shortcomings of the same type of research. First of all, the measurement instrument currently used was developed and designed by the researchers themselves. Therefore, developing and applying more targeted scales to assess teaching quality and student satisfaction with teaching should be advanced in the future. Meanwhile, the representativeness of this research study may be lacking as only two classes of students from the same University were selected as the study subjects. Since the study subjects of the PBL group and traditional group are not in the same class, the assessment standards and the difficulty of the test paper are different, so the two groups’ age and grade point average are statistically different, which may affect the interpretation of the results. In addition, the small sample size is another limitation. This may bias the results and reduce the transferability of the study results. The sample size could be further expanded, and the observation indicators could be enriched in the follow-up study better to explore the clinical advantages of this teaching method. Finally, the questionnaire was subject to information bias. Cross-sectional research and prospective cohort studies with multiple regions, various types of schools, and large samples should be the goal and direction of the following study.

## Conclusion

In summary, the online teaching of Tencent conferences based on the PBL method can improve students’ participation, teacher-student interaction, acceptance, and understanding of the internship process to a certain extent. In addition, compared with the traditional teaching mode, implementing the PBL method can cultivate medical students’ clinical questioning and examination ability and significantly improve their ability to analyse and solve problems. However, whether this experiment has any effect on shaping physicians’ sense of professionalism needs to be further explored. Clinical internship is a vital part of clinical teaching in cardiac surgery. However, the current internship model is mainly based on traditional lectures, which only superficially affect the students’ knowledge and understanding. How to improve student’s mastery and application of theoretical knowledge in cardiac surgery is an issue that every teacher should focus on. Future educators should consider using the PBL teaching method flexibly alone or in combination with other techniques depending on the content and in conjunction with analysing the learning situation, thus providing students with an environment that promotes intrinsic learning motivation and simultaneously improves their overall quality.

## Supporting information

S1 Raw data The raw data. (XLSX)
